# Response: Rectus Sheath Hematoma Can Resemble Bladder Hematoma on Ultrasound

**DOI:** 10.31662/jmaj.2023-0140

**Published:** 2023-11-16

**Authors:** Hiroshi Shiba, Tomomi Endo, Hirohisa Fujikawa, Tatsuo Tsukamoto

**Affiliations:** 1 Department of Nephrology and Dialysis, Medical Research Institute KITANO HOSPITAL, PIIF Tazuke-Kofukai, Osaka, Japan; 2 Department of Internal Medicine, Suwa Central Hospital, Nagano, Japan; 3 Department of Medical Education Studies, International Research Center for Medical Education, Graduate School of Medicine, The University of Tokyo, Tokyo, Japan

**Keywords:** rectus sheath hematoma, coronavirus disease 2019, bladder hematoma, Fothergill, peritoneal sign

## To the Editor:

We thank Drs. Steven, Haili, and Eileen’s insightful comments on our case report. We appreciate the opportunity to respond to the points raised in the letter and provide further clarification.

First, it has been suggested that patients infected with coronavirus disease 2019 (COVID-19) may be at risk of bleeding. Additionally, iatrogenic factors, including anticoagulation, contribute to hemorrhage. However, thrombocytopenia, reduced fibrinogen, and disseminated intravascular coagulation complicated with COVID-19 are associated with bleeding ^(1)^. Özer et al. have reported a higher incidence of spontaneous rectus sheath hematoma (RSH) in patients with COVID-19 than cases of acute abdominal pain ^(2)^. In this case series, anticoagulation may not be the primary cause of RSH because the activated partial thromboplastin time values remained within the normal range. While the potential influence of coughing has not been adjusted, we cannot deny that vasculopathy due to COVID-19 may play a role in RSH.

Second, the physical signs of RSH vary based on its size. As authors have pointed out, Fothergill’s sign is useful when the abdominal wall mass is not large. In contrast, RSH extending into the peritoneum and prevesical space, often classified as type III, can present with peritoneal signs. Furthermore, Cherry et al. reported peritoneal irritation was observed in 9.5% of RSH cases ^(3)^. In our sarcopenic patient, the large hematoma crossed the midline and extended deeply into the peritoneal cavity.

Third, according to the authors’ comment, we would like to refer to the image as “pseudo bladder hematoma” rather than “pseudo bladder sign.” This terminology is more appropriate because distinguishing between RSH and urinary bladder hemorrhage can be challenging on ultrasound when both show hypoechoic fluid collection over a hyperechoic layer ^(4)^.

Therefore, we conclude that the patient developed RSH attributable to COVID-19, which was large enough to exhibit peritoneal signs and resemble a bladder hemorrhage on ultrasound. Further accumulation of case series is required to clarify the causal relationship.

## Article Information

### Conflicts of Interest

None

### Author Contributions

HS drafted the manuscript. TE, HF, and TT reviewed and supervised the manuscript.

### Informed Consent

Not applicable

### Approval by Institutional Review Board (IRB)

In this study, IRB approval was not required.
